# Lung cancer and women: results of a French case-control study.

**DOI:** 10.1038/bjc.1987.19

**Published:** 1987-01

**Authors:** E. Benhamou, S. Benhamou, R. Flamant

## Abstract

Ninety-six women with histologically confirmed lung cancer and 192 matched controls were involved in an international case-control study conducted from 1976 to 1980. The aim of this study was an examination of the effects of different smoking habits, especially the type of cigarettes smoked (light or dark tobacco and filter or nonfilter use) on the occurrence of lung cancer in French females. All these patients were either nonsmokers or lifetime cigarette smokers. Matched relative risk (RR) of smokers compared to nonsmokers was found to be increased for both Kreyberg I (RR = 6.6) and Kreyberg II (RR = 2.1) categories; however, this increase was significant (P less than 0.0001) only for Kreyberg I lung cancer. A significant increase (P less than 0.0001) in matched RR was found with early age at first cigarette smoked, daily consumption, duration of smoking, frequency of inhalation, use of dark tobacco and use of nonfilter cigarettes. Matched RR associated with smokers not always using dark tobacco and those smoking only dark tobacco as compared to nonsmokers were significantly increased (trend test P less than 0.0001). On the contrary, the increase of RR was not significant when either daily consumption, or duration of smoking, or age at first cigarette was taken into account. Lung cancer appeared to be associated with daily consumption and use of nonfilter cigarettes in a matched logistic regression.


Br. J. Cancer (1987), 55, 91–95 © The Macmillan Press Ltd., 1987

### Lung cancer and women: Results of a French case-control study

E. Benhamou[1], S. Benhamou[2] & R. Flamant[1,2]

[1]*Département de Statistique Médicale and* [2]*Unité de Recherches en Epidémiologie des Cancers de l'INSERM (U 287), Institut Gustave Roussy, 94805 Villejuif Cedex, France.*

**Summary** Ninety-six women with histologically confirmed lung cancer and 192 matched controls were involved in an international case-control study conducted from 1976 to 1980. The aim of this study was an examination of the effects of different smoking habits, especially the type of cigarettes smoked (light or dark tobacco and filter or nonfilter use) on the occurrence of lung cancer in French females. All these patients were either nonsmokers or lifetime cigarette smokers. Matched relative risk (RR) of smokers compared to nonsmokers was found to be increased for both Kreyberg I (RR = 6.6) and Kreyberg II (RR = 2.1) categories; however, this increase was significant ($P < 0.0001$) only for Kreyberg I lung cancer. A significant increase ($P < 0.0001$) in matched RR was found with early age at first cigarette smoked, daily consumption, duration of smoking, frequency of inhalation, use of dark tobacco and use of nonfilter cigarettes. Matched RR associated with smokers not always using dark tobacco and those smoking only dark tobacco as compared to nonsmokers were significantly increased (trend test $P < 0.0001$). On the contrary, the increase of RR was not significant when either daily consumption, or duration of smoking, or age at first cigarette was taken into account. Lung cancer appeared to be associated with daily consumption and use of nonfilter cigarettes in a matched logistic regression.

The incidence of lung cancer among women is increasing in most industrialized countries. In recent years, the rate of increase among women has been greater than among men. However, the mortality rate of lung cancer among males is still very much higher than in females: for instance, in the United States, it has been reported to be twice as high as in females (Starzik, 1983). Most authors concur in the belief that, if this trend persists in the United States, lung cancer will be the major cause of death by cancer among women, instead of breast cancer – as observed now in California, Washington and Louisiana (Loeb *et al.*, 1984) – and lung cancer death rates for females may equal those for males by the year 2000 (Starzik, 1983). This evolution is associated with an increase in cigarette consumption among females over the past 20 to 30 years (Fraumeni & Blot, 1982). The situation in France is, however, unusual insofar as lung cancer mortality in females is low compared to other industrialized countries (Hirohata *et al.*, 1982) and increased only very slightly between 1952 and 1982 (INSERM, 1980; 1984).

A large number of epidemiologic studies on lung cancer have been reviewed in two reports of the Public Health Service (Office on smoking and health, 1980; 1982). Over the past thirty years, the association between lung cancer and tobacco consumption (principally cigarettes) among males has been demonstrated, and in some recent investigations among females, lung cancer and the use of tobacco have also been found to be associated, though less strongly (Office on smoking and health, 1980).

This paper reports the results observed among females of a case-control study, undertaken to evaluate the role of cigarette smoking habits, especially the type of cigarettes smoked (dark or light tobacco and filter or nonfilter use) in the causation of lung cancer.

### Materials and methods

An epidemiologic study on lung cancer was conducted simultaneously in France and in four other European countries Austria, Germany, Italy (Milan, Rome), and Scotland with the support of the US National Cancer Institute. In France, this case-control study was performed from 1976 to 1980. A total of 1,625 cases with histologically confirmed lung cancer and 3091 controls whose current diseases were not tobacco-related were included. Each case was matched with two controls for sex, age at diagnosis, hospital of admission and interviewer. A complete description of this study can be found in previous papers (Lubin *et al.*, 1984; Benhamou *et al.*, 1985). The results presented here are those observed in females; that is, 96 cases and 192 matched controls.

Of these 96 cases, 50 cancers were squamous (52%), 16, undifferentiated (17%), 24, adenocarcinoma (25%) and 6, unspecified (6%). The first two histological categories (squamous and undifferentiated) constitute the Kreyberg I category (66 cases), and the third (adenocarcinoma type) constitutes the Kreyberg II category (24 cases).

Among the 192 controls, the main diagnostic categories were: bone diseases (21%), malignant tumours (14%) excluding respiratory tract, oesophagus, liver, pancreas, bladder and kidney, trauma (13%), viral and other infective diseases (8%), benign tumours (4%) and neurological diseases (3%).

### *Analytical method*

Adjusted RRs of lung cancer were estimated using the Mantel–Haenszel method (Mantel, 1963), and 95% CI with the use of the Cornfield (1956) method. The different parameters characterising the smoking habits have been analysed by a logistic regression (Breslow & Day, 1980) taking into account the full matching of each case with her two original controls. In order not to eliminate matched stratas, nonsmokers were retained in the analysis. The coding used allows the definition of all possible combinations of cigarette exposure, and the definition of the referent category (nonsmokers) by combining nonsmokers with the lowest level of exposure for three of the four variables used.

### Results

Mean age at diagnosis and educational level do not differ significantly either between cases and controls, or between Kreyberg I and Kreyberg II cases (Table I).

### *Smoking habits*

All smoking cases and controls used only cigarettes. The

Correspondence: E. Benhamou.
Received 22 January 1986; and in revised form, 14 August 1986.

**Table I** Distribution of Kreyberg I and Kreyberg II lung cancer cases and their controls by age at diagnosis and educational level

| | *Kreyberg I* (66) % | *Controls* (132) % | *Kreyberg II* (24) % | *Controls* (48) % |
|---|---|---|---|---|
| Age at diagnosis (years) | | | | |
| <40 | 1 | 2 | 0 | 2 |
| 40–49 | 11 | 13 | 21 | 17 |
| 50–59 | 39 | 38 | 29 | 33 |
| 60–69 | 29 | 25 | 29 | 29 |
| ≧70 | 20 | 22 | 21 | 19 |
| Mean age (±2 s.e.) | 59.3 (2.4) | 59.7 (1.8) | 59.9 (4.2) | 59.4 (3.1) |
| Education (years) | | | | |
| <8 | 61 | 62 | 62 | 69 |
| 8–13 | 36 | 30 | 38 | 27 |
| ≧14 | 3 | 8 | 0 | 4 |
| Mean years (±2 s.e.) | 7.0 (0.9) | 6.9 (0.6) | 5.8 (1.4) | 6.2 (0.9) |

percentage of regular smokers, i.e. people having smoked at least one cigarette per day for at least one year was, of course, significantly greater ($P<0.0001$) among cases (48%) than among controls (17%). It was, moreover, significantly higher ($P<0.05$) in Kreyberg I cases (55%) than in Kreyberg II cases (29%). The matched RR of smokers relative to nonsmokers in the Kreyberg I category was 6.6 ($P<0.0001$). On the contrary, in the Kreyberg II category, the excess of risk for smokers *versus* nonsmokers (RR = 2.1) was not significant, probably due to the low number of cases (Table II).

Separate study of the different parameters measuring the exposure to cigarettes, among the 96 cases and 192 matched controls, showed an increased matched risk in smokers compared to nonsmokers for the following parameters: age at first cigarette, daily consumption, frequency of inhalation and duration of smoking (Table III). Trend tests were highly significant for each of these four variables ($P<0.0001$).

In this study, 58% smokers smoked dark tobacco exclusively. Three categories of smokers were defined: the first comprised those having smoked dark tobacco for half

**Table II** Matched RR of lung cancer of smokers to nonsmokers

| | *Kreyberg I* | | | | *Kreyberg II* | | | |
|---|---|---|---|---|---|---|---|---|
| | *Cases* | *Controls* | *RR*[a] | *95% CI* | *Cases* | *Controls* | *RR*[a] | *95% CI* |
| Nonsmokers | 30 | 109 | 1.0 | | 17 | 41 | 1.0 | |
| Smokers | 36 | 23 | 6.6[b] | 3.0–14.4 | 7 | 7 | 2.1 | 0.7–6.4 |

[a]All matched RR were calculated *versus* nonsmokers. [b]$P<0.0001$.

**Table III** Matched RR of lung cancer according to variables characterizing cigarette consumption

| | *Cases* | *Controls* | *RR*[a] *(95% CI)* | *Trend test P-value* |
|---|---|---|---|---|
| Nonsmokers | 50 | 159 | 1.00 | |
| Age at first cigarette smoked (yrs) | | | | <0.0001 |
| >30 | 4 | 6 | 1.77 (0.48–6.55) | |
| 21–30 | 15 | 13 | 3.72 (1.61–8.58) | |
| ≦20 | 27 | 14 | 8.16 (3.99–19.64) | |
| No. of cigarettes smoked per day | | | | <0.0001 |
| <10 | 5 | 14 | 1.23 (0.41–3.73) | |
| 10–19 | 11 | 11 | 2.88 (1.16–7.15) | |
| ≧20 | 30 | 8 | 19.97 (5.96–66.93) | |
| Duration of smoking (yrs) | | | | <0.0001 |
| 1–20 | 5 | 13 | 1.17 (0.34–3.62) | |
| 21–40 | 28 | 15 | 6.21 (2.79–13.82) | |
| ≧41 | 13 | 5 | 9.45 (2.62–34.17) | |
| Inhalation | | | | <0.0001 |
| No | 15 | 18 | 2.80 (1.25–6.28) | |
| Yes | 31 | 15 | 6.58 (3.11–13.94) | |
| Type of tobacco | | | | <0.0001 |
| ≦50% dark | 12 | 13 | 2.87 (1.20–6.89) | |
| 51–99% dark | 4 | 4 | 4.77 (1.06–21.42) | |
| 100% dark | 30 | 16 | 6.10 (2.91–12.77) | |
| Use of filter | | | | <0.0001 |
| ≦50% non filter | 17 | 20 | 2.54 (1.21–5.31) | |
| 51–99% non filter | 12 | 9 | 7.15 (2.25–22.77) | |
| 100% non filter | 17 | 4 | 16.01 (4.72–54.33) | |

[a]All matched RR were calculated *versus* nonsmokers.

of their tobacco history or less (≦50% dark); the second, those having smoked dark tobacco for more than half of their tobacco history (>50% dark) and the last, those having never smoked anything but dark tobacco (100% dark). Similarly, considering the use of filters, three categories were defined: the first comprised those having smoked nonfilter cigarettes for half of their tobacco history or less (≦50% nonfilter); the second, those having smoked nonfilter cigarettes for more than half of their tobacco history (>50% nonfilter), and the last, those having always smoked nonfilter cigarettes (100% nonfilter). A significant increase ($P<0.0001$) of lung cancer matched risk was found with the type of tobacco smoked and with the use of nonfilter cigarettes (Table III). An increased risk, although not significant, was found for 100% dark tobacco *versus* ≦50% dark tobacco users (RR = 2.04) after adjustment for age. On the contrary, the excess of risk for 100% nonfilter *versus* ≦50% nonfilter users (RR = 4.44) was significant ($P<0.03$).

All the parameters described above were studied together in a matched logistic model. This method allowed the estimation of RR for each variable when adjusting on the others. Table IV shows a significant excess of risk associated with daily consumption ($P<0.03$) and use of nonfilter cigarettes ($P<0.06$). An increase of risk, although not significant, was found with duration of smoking and frequency of inhalation. The significance of age at first cigarette and type of tobacco disappeared as soon as duration was introduced into the model, so that these two covariates were not taken into account in the final model. However, the small number of cases and controls, associated with the strong correlations of the smoking-related-variables do not allow a clear interpretation of these results. The effect of the type of tobacco was studied using a matched logistic regression (Table V). Two categories of tobacco smokers were defined (100% dark tobacco users or not). RR associated with each category of smokers as compared to nonsmokers were significantly increased (trend test $P<0.0001$). On the contrary, the increase of RRs were not significant when either daily consumption or duration of smoking, or age at first cigarette was taken into account.

Among smokers, 19.5% cases and 27.3% controls were ex-smokers. The excess of risk of current smokers to ex-smokers was not statistically significant and though the

**Table IV** Results of the multivariate analysis of characteristic parameter of smoking habits in cases and matched controls

| *Variables* | *Log likelihood* | *RR (95% CI)* | *P value (trend)* |
|---|---|---|---|
| No variable | −105.47 | | |
| All variables | −81.84 | | |
| No. of cigarettes/day | −83.72[a] | | <0.03 |
| Nonsmokers | | 1.00 | |
| <10 | | 0.57 (0.11–2.59) | |
| 10–19 | | 0.89 (0.18–4.44) | |
| ≧20 | | 4.85 (0.66–35.64) | |
| Use of filter | −83.15[a] | | <0.06 |
| ≦50% nonfilter[b] | | 1.00 | |
| >50% nonfilter | | 1.27 (0.27–5.95) | |
| 100 nonfilter | | 3.62 (0.68–19.18) | |
| Duration of smoking, yr | −82.03[a] | | NS |
| ≦20[b] | | 1.00 | |
| 21–40 | | 2.10 (0.49–8.96) | |
| >40 | | 3.27 (0.44–24.14) | |
| Inhalation | −81.87[a] | | NS |
| Never[b] | | 1.00 | |
| Mostly or always | | 1.54 (0.44–5.45) | |

[a]Value represents the log likelihood function when the corresponding covariate is removed from the all variables model. [b]The less exposed category includes nonsmokers.

*Footnote* Considering nonsmokers as referent category for each of the 4 variables, the regression model is overparametrised. The coding used allows the definition of all possible and logical combinations of cigarette exposure, and the definition of the referent category (nonsmokers) by combining nonsmokers with the lowest level of exposure for 3 of the 4 variables introduced in the model. For each covariate, the risk presented in the Table, adjusted on the other covariates, is estimates *versus* the defined referent category. However, in the risk function, as soon as cigarette consumption defined *versus* nonsmokers is introduced, the estimated risk is calculated *versus* nonsmokers.

**Table V** Matched RR (95% CI) of lung cancer by type of cigarettes smoked

| | *RR* | *RR adjusted on daily consumption* | *RR adjusted on duration* | *RR adjusted on age at first cigarette* |
|---|---|---|---|---|
| Nonsmokers | 1.00 | 1.00 | 1.00 | 1.00 |
| Not always dark | 3.20 (1.43–7.16) | 1.06 (0.36–3.07) | 1.32 (0.44–3.88) | 1.56 (0.51–4.80) |
| Always dark | 5.94 (2.86–12.31) | 0.74 (0.19–2.86) | 1.86 (0.58–5.95) | 2.21 (0.62–7.88) |
| Trend test | <0.0001 | NS | NS | NS |

number of years since cessation is greater among controls (11.4) than among cases (5.1), the difference was not significant.

### Discussion

General critical comments on the protocol of this international case-control study have been presented in recent papers on the total international data (Lubin *et al.*, 1984) and about French data in men (Benhamou *et al.*, 1985).

Smoking-related variables found to be significantly associated with Kreyberg I, were also found to increase the risk of adenocarcinoma (Lubin & Blot, 1984). In this study, the association between cigarette smoking habits and lung cancer among females was found to be significant ($P<0.0001$) for the Kreyberg I category. The small number of women with adenocarcinoma can explain the lack of significant difference in the association between lung cancer and cigarette exposure.

The analysis of the different measures of exposure to cigarettes need to be interpreted cautiously because of the relatively small number of women. However, because of the low incidence of female lung cancer in French women, the same amount of time was necessary to include these 96 cases as was needed for the 1,529 male lung cancer cases of this study. This low number of cases is likely to explain why risks associated with classical variables such as duration of cigarettes, although increased, were not significant in the multivariate analysis; moreover, proper effects of type of tobacco and use of filter could not be evaluated simultaneously. In spite of these reservations, the results observed for filter use are consistent in matched univariate analysis and matched logistic regression, i.e. a more harmful effect of nonfilter compared to filter use. The effect of dark compared to light tobacco, though increased, is not significant, probably because of the small number of patients smoking light tobacco.

The usual differences reported in the distribution of the different histologic types of cancer among females, compared with those observed among males (Office on smoking and health, 1980, 1982; Lubin & Blot, 1984) are also found in our study: the percentage of Kreyberg I cases was greater among males (82%) than among females (69%); and, contrary to this, Kreyberg II lung cancer was more frequent among females (25%) than among males (9%). Also, as described in other studies (Office on smoking and health, 1980, 1982), within each histologic type, the percentages of smokers are quite different: in our study, among males and females, the percentages of nonsmokers among adenocarcinoma (8% and 71% resp.) are greater than among Kreyberg I cases (2% and 45% resp.).

The results of this study confirm those of the literature, i.e. a stronger association between lung cancer and cigarette smoking among males than among females, and among Kreyberg I than among adenocarcinoma. However, the lower risk of lung cancer observed for women than for men, must be interpreted cautiously. Indeed, in our sample, although the mean age at diagnosis was similar among male and female cases, tobacco habits were very different for male and female populations with Kreyberg I lung cancer. Among smokers, the average age at first cigarette among women (23 yrs) was significantly greater ($P<0.001$) than among men (19 yrs). The average number of cigarettes smoked per day among women was 22, and the average duration of the smoking habit was 34 years. These figures were lower than those observed for men (24 cigarettes per day, and 38 yrs resp.). Similarly, for women with lung cancer, the percentage of subjects who inhaled deeply was 36%, the percentage of lifetime dark tobacco smokers was 69% and the percentage of exclusive nonfilter cigarettes smokers was 33%. For men, these percentages were higher (37%, 91%, 65% resp.).

Thus, the lower risk of Kreyberg I lung cancer for women *versus* men could be partially explained by these differences in smoking habits. However, the substantial percentage of nonsmokers among women with lung cancer, especially adenocarcinoma, is consistent with the implication of risk factors such as hormones exclusive to women (Lubin & Blot, 1984).

Concerning lung cancer in women, the situation in France is very special, since lung cancer mortality in females is low compared to other industrialized countries (Hirohata *et al.*, 1982) and has increased only very slightly between 1952 and 1982. This can be explained by later smoking patterns in French women compared, for instance, to American women and we can expect a higher frequency of lung cancer in French women over the next few years.

Existence of a 25 to 30 year interval before the marked increase in consumption of cigarettes by women as compared to men (Hill & Flamant, 1985), suggests that current figures may not yet constitute, particularly in France, a demonstration of the maximal health effects of smoking in women.

Supported by Public Health Service contract N01CP-05642 from the Division of Cancer Cause and Prevention, National Cancer Institute.

We thank the National Cancer Institute for its support and Dr E.L. Wynder for the implementation of the protocol. We are indebted to Drs R. Arriagada, J.P. Bader, G. Batesti, J. Bignon, P. Bilski-Pasquier, H. Bismuth, C. Blatrix, P. Bouche, B. Bour, C. Boutin, J. Brissard, M. Camey, I. Caubarrere, J. Chretien, D. Chassagne, A. Chavy, P. Choubrac, A. Cornet, B. Court, J. Courty, V. Demassieux, G. De Ren, N. De Saint Florent, J. Dormont, P. Duroux, J. Guedon, J.P. Kleisbauer, J. Lacour, P. Lamy, T. Le Chevalier, G. Lemoine, E. Letournel, G. Manigand, J. Marsac, P. Massias, G. Mathe, A. Monsaingeon, P. Morin, R. Pariente, C. Perol, G. Pierard, J. Pointillard, J. Rainaut, S. Redon, J. Rochemaure, J. Sebaoun, D. Silbert, C. Sors & G. Vergez, for their contributions to data. We are grateful to S. Fallah for her technical assistance.

### References

BENHAMOU, S., BENHAMOU, E., TIRMARCHE, M. & FLAMANT, R. (1985). Lung cancer and use of cigarettes: A French case-control study. *J. Natl Cancer Inst.*, **74,** 1169.

BRESLOW, N.E. & DAY, N.E. (1980). Statistical methods in cancer research. Vol. 1. The analysis of case-control studies. *IARC Sci. Publ.*, **32.**

CORNFIELD, J. (1956). A statistical problem arising from retrospective studies. In *Proceedings of the Third Berkeley Symposium*, IV, Neyman, J. (ed). University of California Press, Berkeley.

FRAUMENI, J.F. & BLOT, W.J. (1982). Lung and pleura. In *Cancer Epidemiology and Prevention*, Schottenfeld, D. & Fraumeni, J.F. (eds). W.B. Saunders Company.

HILL, C. & FLAMANT, R. (1985). Une cause d'épidémie majeure: l'augmentation de la consommation de tabac en France. *Rev. Epidém. et Santé Publ.*, **33,** 387.

HIROHATA, T., YOSHIDA, A. & SHIBATA, A. (1982). Age-adjusted death rates of malignant neoplasms of various sites for 33 selected countries in the World. *Kurume Med. J.*, **29,** 1.

INSERM (1980). Le Cancer, la mortalité en 1976, son évolution depuis 1954. INSERM, Paris.

INSERM (1984). Statistique des causes médicales de décès de 1977 à 1982. INSERM, Paris.

LOEB, L.A., ERNSTER, V.L., WARNER, K.E., ABBOTTS, J. & LASZLO, J. (1984). Smoking and lung cancer: An overview. *Cancer Res.*, **44,** 5940.

LUBIN, J.H., BLOT, W.J., BERRINO, F. & 5 others (1984). Patterns of lung cancer risk according to type of cigarette smoked. *Int. J. Cancer,* **33,** 569.

LUBIN, J.H. & BLOT, W.J. (1984). Assessment of lung cancer risk factors by histologic category. *J. Natl Cancer Inst.,* **73,** 383.

MANTEL, N. (1963). Chi-square test with one degree of freedom; extension of the Mantel–Haenszel procedure. *J. Amer. Stat. Assoc.,* **58,** 690.

OFFICE ON SMOKING AND HEALTH (1980). The health consequences of smoking for women. A report of the Surgeon General. Public Health Service, US Department of Health and Human Services. Rockville, Maryland.

OFFICE ON SMOKING AND HEALTH (1982). The health consequences of smoking: Cancer. A report of the Surgeon General. Public Health Service. US Department of Health and Human Services, Rockville, Maryland.

STARZIK, P.M. (1983). Lung-cancer deaths: equality by 2000? *N. Engl. J. Med.,* **308,** 1289.


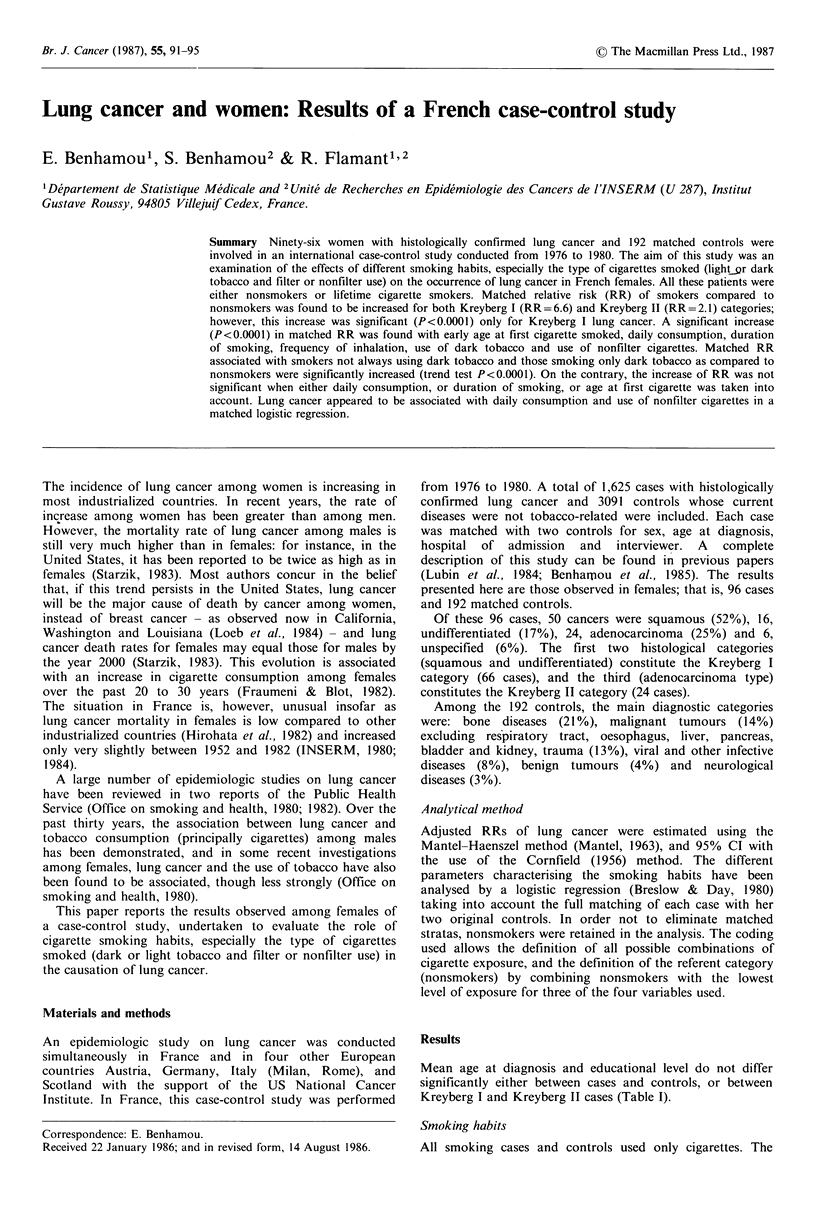

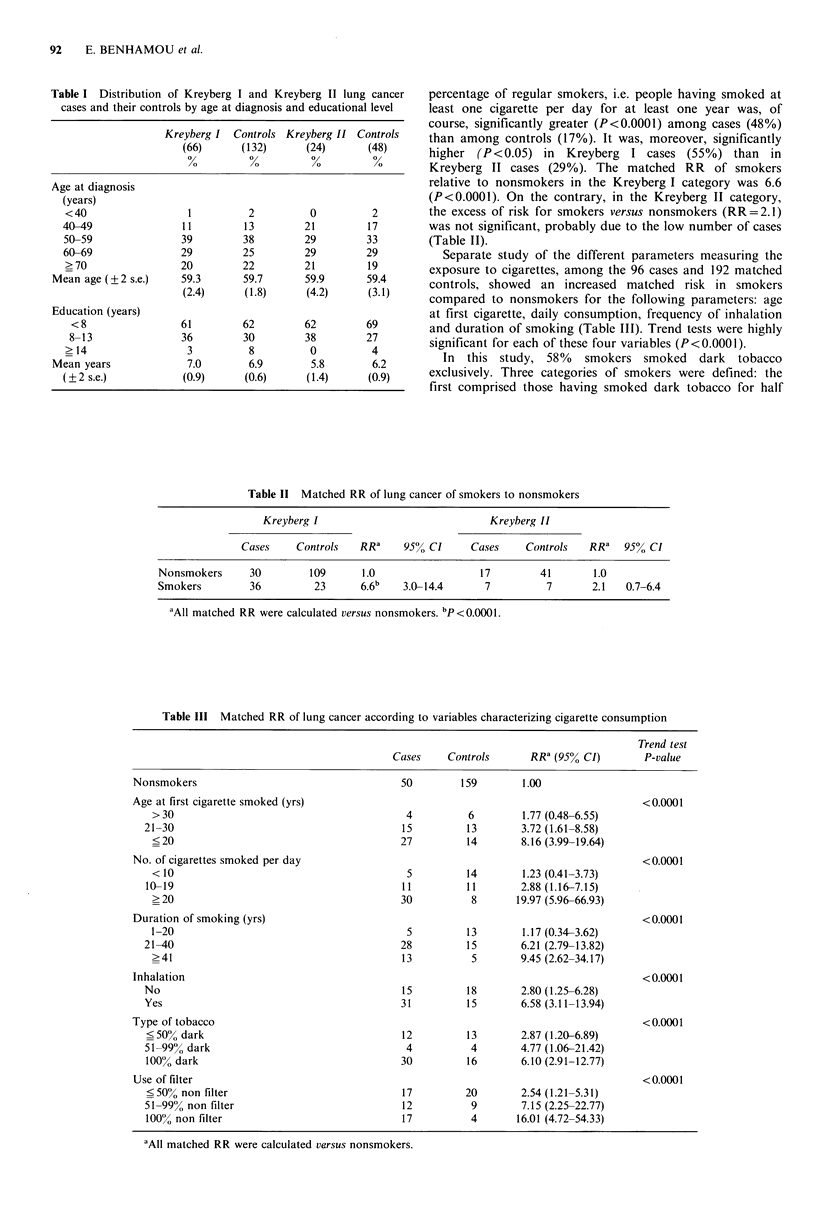

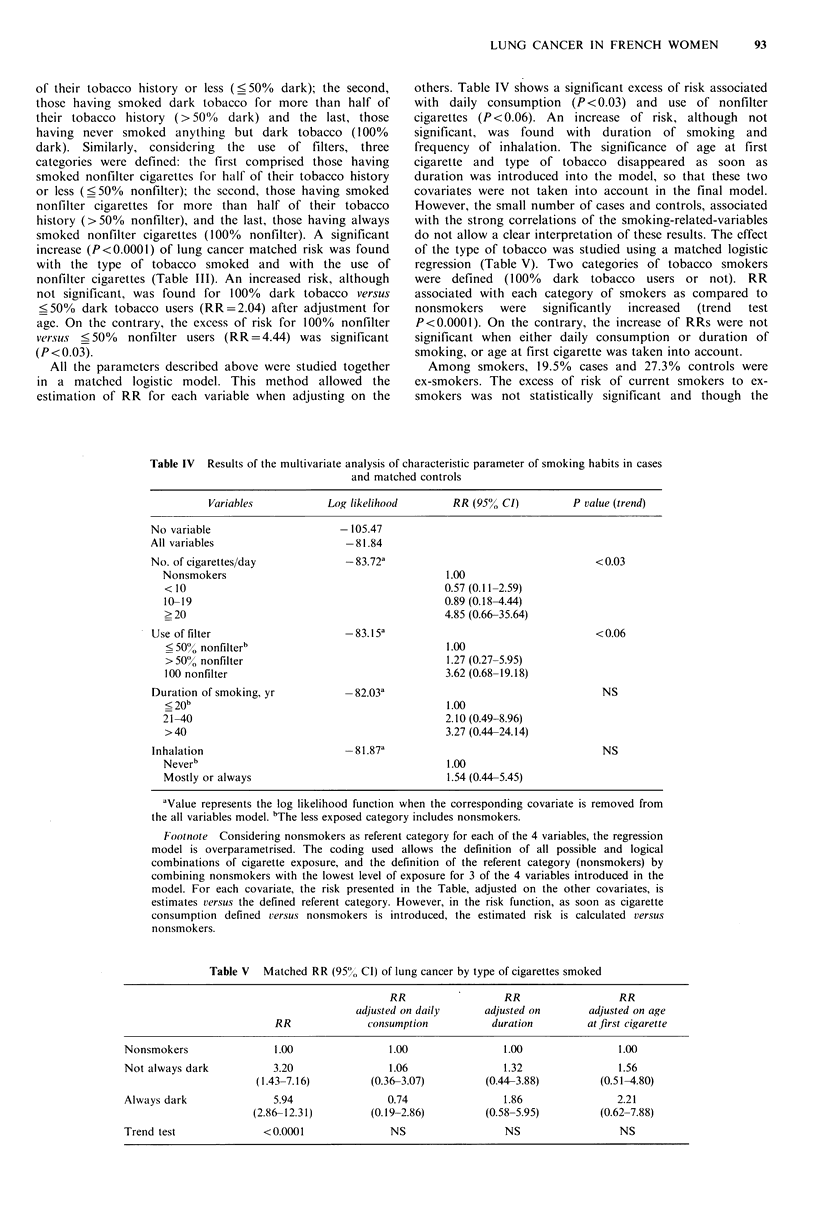

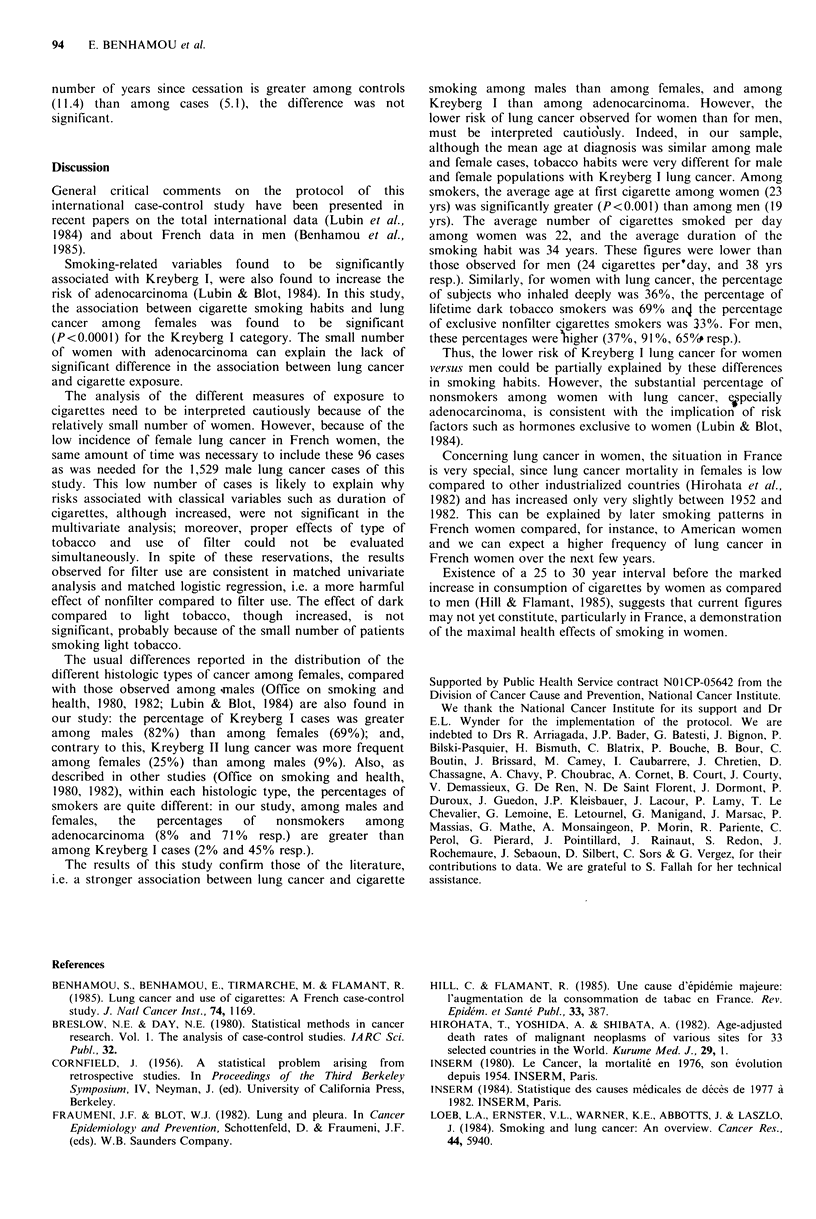

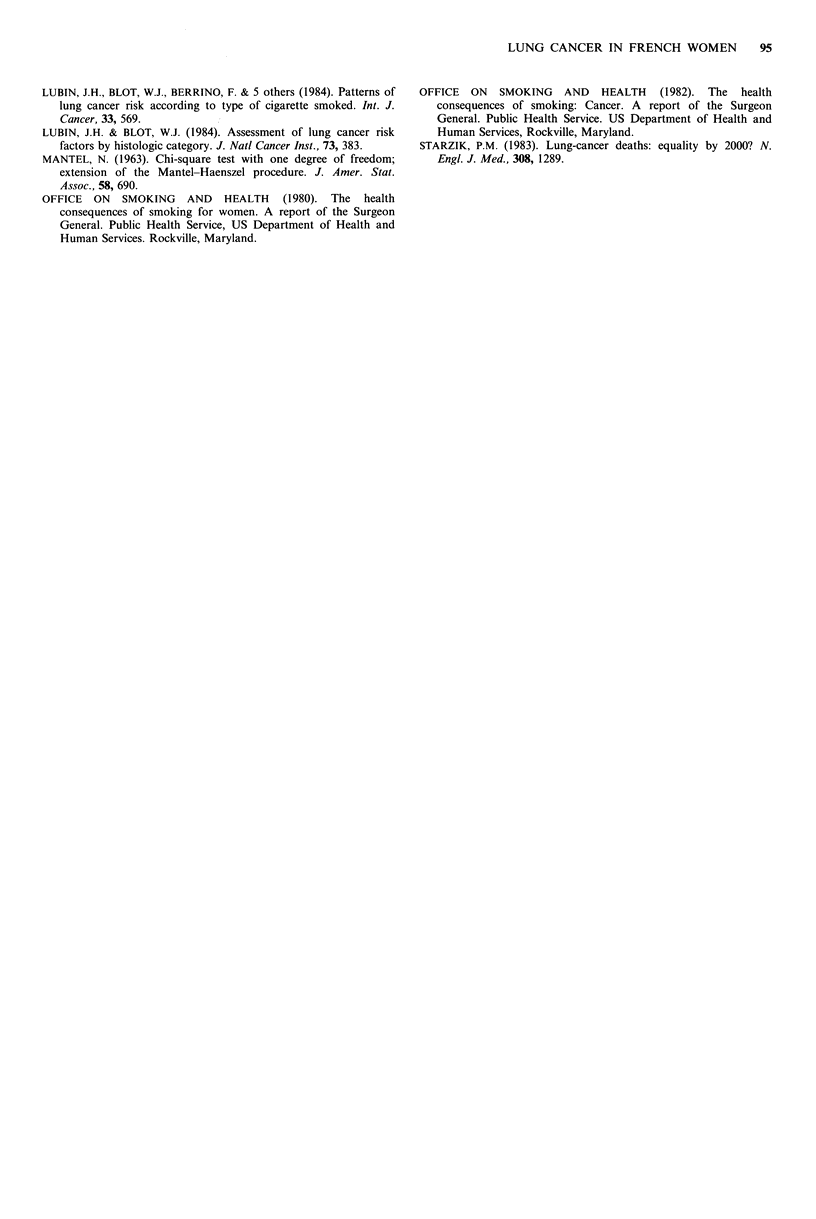


## References

[OCR_00486] Benhamou S., Benhamou E., Tirmarche M., Flamant R. (1985). Lung cancer and use of cigarettes: a French case-control study.. J Natl Cancer Inst.

[OCR_00507] Hill C., Flamant R. (1985). Une cause d'épidémie majeure: l'augmentation de la consommation de tabac en France.. Rev Epidemiol Sante Publique.

[OCR_00512] Hirohata T., Yoshida A., Shibata A. (1982). Age-adjusted death rates of malignant neoplasms of various sites for 33 selected countries in the world.. Kurume Med J.

[OCR_00525] Loeb L. A., Ernster V. L., Warner K. E., Abbotts J., Laszlo J. (1984). Smoking and lung cancer: an overview.. Cancer Res.

[OCR_00537] Lubin J. H., Blot W. J. (1984). Assessment of lung cancer risk factors by histologic category.. J Natl Cancer Inst.

[OCR_00532] Lubin J. H., Blot W. J., Berrino F., Flamant R., Gillis C. R., Kunze M., Schmahl D., Visco G. (1984). Patterns of lung cancer risk according to type of cigarette smoked.. Int J Cancer.

[OCR_00558] Starzyk P. M. (1983). Lung-cancer deaths: equality by 2000?. N Engl J Med.

